# Intravascular Imaging to Predict the Coronary No-Reflow and Slow-Flow Phenomena: Advances and Perspectives

**DOI:** 10.31083/RCM47922

**Published:** 2026-08-18

**Authors:** Yaokun Zhan, Qili Huang, Qingkuan Li, Huayuan Zeng, Ying Shi, Zhengde Lu, Huili Li, Junpeng Xiao, Hongqing Li, Yingzhong Lin, Qingwei Ji

**Affiliations:** ^1^Department of Cardiology, Guangxi Chest Pain Center, The People’s Hospital of Guangxi Zhuang Autonomous Region, and Institute of Cardiovascular Diseases, Guangxi Academy of Medical Sciences, 530000 Nanning, Guangxi, China

**Keywords:** no-reflow, intravascular ultrasound, optical coherence tomography, near-infrared spectroscopy

## Abstract

Coronary no-reflow (NF) and slow-flow (SF) are serious complications following percutaneous coronary intervention (PCI) and are associated with adverse prognoses, with microvascular obstruction (MVO) as the central underlying mechanism. Intravascular imaging techniques, including intravascular ultrasound (IVUS), optical coherence tomography (OCT), and near-infrared spectroscopy (NIRS), enable high-resolution, multidimensional (structural, compositional, and functional) assessment of coronary lesions, thereby facilitating prediction of coronary NF and SF. IVUS, with a penetration depth of 4–8 mm, can identify predictive features such as plaque rupture, attenuated plaque, positive remodeling, and large necrotic cores. OCT, with a spatial resolution of 10–20 μm and a penetration depth of 1–2 mm, is capable of detecting thin-cap fibroatheromas, cholesterol crystals, and intraplaque vasa vasorum. NIRS quantifies lipid content using the parameter maximum 4 mm lipid core burden index (maxLCBI_4mm_), which demonstrates favorable predictive performance for NF and SF phenomena. When the OCT-derived fibrous cap thickness is <55 µm, the area under the curve (AUC) for predicting NF is 0.985 (95% confidence interval (CI): 0.968–1.000); meanwhile, the corresponding sensitivity and specificity for predicting SF are 93.5% and 96.7%, respectively. When the NIRS-derived maxLCBI_4mm_ is ≥578, the AUC for predicting SF is 0.849.

## 1. Introduction

Coronary no-reflow (NF) and slow-flow (SF) phenomena are common and severe complications following percutaneous coronary intervention (PCI). A defining pathological feature of these conditions is the failure to achieve effective myocardial perfusion, even when obstruction of the epicardial coronary artery has been successfully resolved by interventional procedures [1]. Under the Thrombolysis in Myocardial Infarction (TIMI) flow grading system—assessed by coronary angiography—NF is defined as TIMI flow grade 0–1 (absent or minimal coronary flow), while SF is defined as TIMI flow grade 2 (delayed flow, with a lower perfusion speed than the physiological level) [2]. Numerous clinical studies have established a strong association between the coronary NF/SF phenomenon and adverse clinical prognoses [2,3,4]. By triggering a cascade of pathological events, these phenomena not only increase the likelihood of myocardial infarction expansion and abnormal left ventricular remodeling but also the occurrence of major adverse cardiovascular events (MACE), such as congestive heart failure and cardiogenic death. Consequently, they significantly compromise the long-term survival prospects of patients undergoing PCI.

From a pathological perspective, the coronary NF/SF phenomenon arises from the coordinated effects of diverse pathogenic factors, with microvascular obstruction (MVO) established as the core mediator linking these factors to clinical manifestations. Key mechanisms underlying MVO include microvascular endothelial impairment triggered by myocardial ischemia, distal vascular embolism resulting from plaque rupture, reperfusion-associated oxidative stress injury, and individual genetic predispositions—all of which act in concert to disrupt microcirculatory integrity [5]. Consequently, the precise preoperative characterization of high-risk lesions and the timely prediction of NF/SF phenomenon risks are critical prerequisites for refining PCI strategies and enhancing long-term patient prognosis.

In recent years, intravascular imaging techniques have emerged as a new paradigm for predicting the coronary NF/SF phenomenon due to their high-resolution visualization of coronary lesions. Intravascular ultrasound (IVUS), optical coherence tomography (OCT), and near-infrared spectroscopy (NIRS) enable multi-dimensional (structural, compositional, functional) analysis of plaques, thereby providing quantitative evidence for clinical decision-making. This article reviews recent research progress on the application of these imaging techniques to predict the coronary NF/SF phenomenon, as well as discussing current technical limitations and likely future directions. The overall aim is to provide useful insights for clinical practice and scientific research.

## 2. Intravascular Imaging Techniques

### 2.1 Intravascular Ultrasound (IVUS)

IVUS operates on the principle of acoustic wave reflection generated by piezoelectric transducers, delivering high-resolution cross-sectional imaging of the coronary lumen and vessel wall. This technology enables precise quantitative assessment of key pathological parameters, including plaque burden, vascular remodeling patterns, and plaque composition [6]. A core advantage of IVUS is its substantial penetration depth (ranging from 4 to 8 mm), which facilitates clear visualization of deep vascular structures such as the external elastic membrane (EEM) and the medial layer. This unique feature renders IVUS particularly well-suited for evaluating complex coronary lesions, such as left main stem, ostial stenoses, bifurcation stenoses, thrombotic lesions, chronically occluded coronary arteries, and calcified coronary artery disease [7]. To date, IVUS has identified a spectrum of lesion characteristics that exhibit strong associations with the development of the coronary NF/SF phenomenon. These are elaborated in detail below (Table 1, Ref. [8,9,10,11,12,13,14,15]).

**Table 1.** **Summarized table of IVUS in predicting coronary no-reflow/slow-fllow phenomenon**.

Marker	Study	Design	Population	Sample size	Cut-off	Sensitivity	Specificity	AUC	OR	95% CI	*p*	Advantages	Limitations
Ruptured plaque cavity volume	Ohshima K et al. [9]	RSC	STEMI	53	≥7.8 mm^3^	78.9%	94.1%	0.862	1.56	1.24–1.96	<0.001	Deep tissue penetration, real-time guidance, no require blood displacement	Resolution may hamper interpretation
AP	Okutsu M et al. [10]	RSC	CAD	707	NP	90.9%	88.9%	NP	NP	NP	NP		
Longitudinal length of AP	Endo M et al. [8]	RSC	STEMI	170	≥5 mm	79%	84%	NP	20.1	5.87–69.0	<0.001		
Longitudinal length of AP	Narayanan S et al. [11]	PMC	ACS	187	≥6.67 mm	69.2%	67.4%	0.675	NP	0.496–0.855	0.037		
Necrotic plaque volume	Utsunomiya M et al. [12]	RSC	IHD	95	≥21.6 mm^3^	81.8%	61.9%	0.766	NP	0.627–0.906	NP		
Ratio of stent diameter to vessel diameter	Watanabe Y et al. [13]	RSC	STEMI	339	0.71	80.4%	56.9%	0.70	2.63	1.84–3.77	<0.001		
Length of plaque prolapse	Narayanan S et al. [11]	PMC	ACS	187	>8.54 mm	81.8%	66%	0.702	1.1	1.0–1.3	0.045		
Estimated LV	Suda A et al. [14]	RSC	CAD	353	≥132.6 mm^3^	68.4%	66.8%	0.719	4.35	1.67–12.7	0.0024		
LV percentage	Daidoji H et al. [15]	RSC	CAD	176	62%	48%	81%	0.67	NP	1.6–13.4	<0.01		

ACS, acute coronary syndrome; AP, attenuated plaque; AUC, area under the curve; CAD, coronary artery disease; CI, confidence interval; IHD, ischemic heart disease; IVUS, intravascular ultrasound; LV, lipid volume; NP, not reported; OR, odds ratio; PMC, prospective multicenter; PSC, prospective single center; RSC, retrospective single center; STEMI, ST-segment elevation myocardial infarction.

#### 2.1.1 Plaque Rupture

**Fig. 1.**
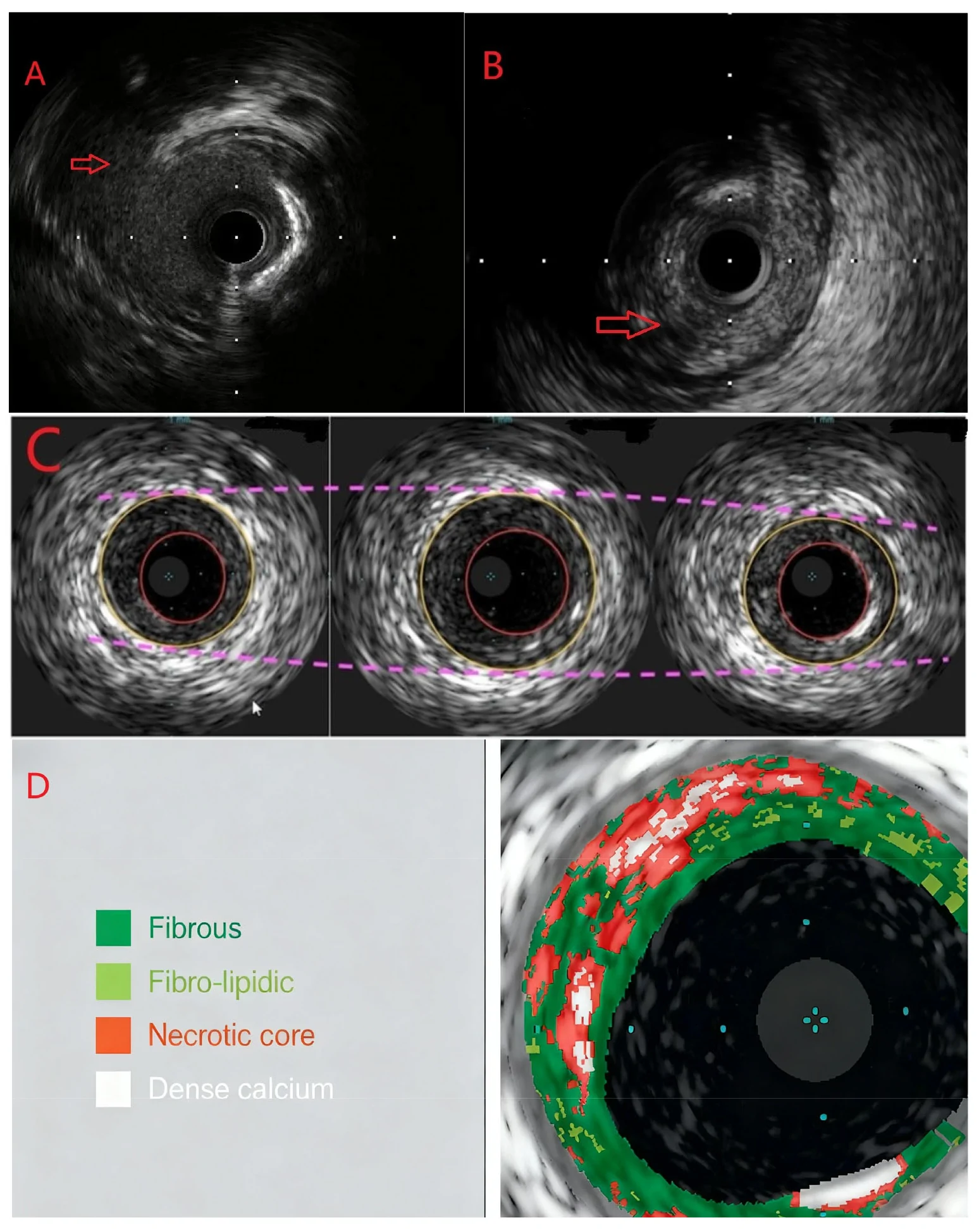
**Lesion characteristics in IVUS to predict coronary no-reflow/slow-flow phenomenon**. (A) Plaque rupture: discontinuity of the fibrous cap (indicated by red arrow). (B) Attenuated plaque: reduced echo intensity within the atherosclerotic region, with no evidence of obvious dense calcification (indicated by red arrow). (C) Positive remodeling: the condition where the ratio of the EEM cross-sectional area at the coronary lesion site to that at the reference segment exceeds 1.05. (D) Necrotic core. EEM, external elastic membrane; IVUS, intravascular ultrasound.

Plaque rupture (PR) is the primary pathological substrate of ST-segment elevation myocardial infarction (STEMI) [16]. On IVUS images, PR is characterized by discontinuity of the fibrous cap. Communication between the rupture site and the coronary lumen can be further confirmed using contrast agents [6] (Fig. 1A). Following PR, procoagulant substances within the lipid core—such as tissue factor and cholesterol crystals (CCs)—are released. These substances readily induce distal embolization and microcirculatory obstruction, ultimately contributing to the development of NF and SF [17]. A prospective study of STEMI patients by Yang et al. [18] found the incidence of NF/SF phenomenon was significantly higher in the PR group than in the non-PR group (26.88% vs. 5.00%, *p* = 0.04). Multivariate analysis further confirmed PR as an independent predictor of NF/SF phenomenon (odds ratio [OR]: 8.188, 95% confidence interval [CI]: 1.020–65.734, *p* = 0.048). The very wide 95% CI of 1.020–65.734 indicates a lack of data precision and limited statistical power, requiring critical interpretation. The lower bound of 1.02 is slightly greater than 1, suggesting a statistically significant positive association between PR and the coronary NF/SF phenomenon. It should be noted, however, that the very wide range of CI (almost 65-fold between the lower and upper bounds) undermines the reliability and clinical interpretability of this association. Such a wide CI implies considerable uncertainty in estimating the true effect size: the actual OR could be as small as approximately 1.02 (a weak association) or as large as 65.734 (an extremely strong association), making it impossible to accurately determine the true strength of the correlation between PR and coronary NF/SF phenomenon. The most plausible underlying cause of a wide CI is insufficient statistical power, which is often attributed to a small sample size or an inadequate number of endpoint events (the coronary NF/SF phenomenon, which is a relatively low-incidence clinical outcome). A small sample size or few events can lead to an increase in the standard error, resulting in unstable regression models and large fluctuations in parameter estimates, even if the point estimate of OR is high. It is crucial to emphasize that statistical significance (implied by the CI not crossing 1) does not equate to reliable or clinically meaningful results. The wide CI suggests the findings may be highly dependent on individual cases, and the OR and its statistical significance could change substantially if a small number of extreme observations are excluded. Additionally, in the context of limited statistical power, any residual confounding, measurement errors, selection bias, and missing data may be disproportionately amplified, further exacerbating the imprecision of the estimate. In summary, while the data suggest a potential association between PR and an increased risk of coronary NF/SF phenomenon, the extremely wide 95% CI indicates the effect size is highly imprecise due to limited statistical power. Hence, the findings of Yang et al. [18] should be regarded as exploratory rather than as reliable evidence for clinical decision-making. Endo et al. [8] also conducted a study of STEMI patients and found an association between PR and an elevated incidence of NF. When multivariate logistic regression analysis was applied, PR emerged as an independent predictor of NF/SF phenomenon (OR: 5.94, 95% CI: 1.64–21.5, *p* < 0.01). A separate study examined patients with acute myocardial infarction (AMI) who received PCI for culprit lesions complicated by PR [19]. This revealed that the presence of multiple PRs, detected via IVUS, was linked to the NF/SF phenomenon in the infarct-related artery. Moreover, multivariate analysis indicated that multiple PRs within the culprit lesion were an independent predictor of NF/SF phenomenon following PCI for the culprit lesion in AMI patients (OR: 15.73, 95% CI: 1.61–153.46, *p* = 0.018).

Further research has underscored the superior predictive value of ruptured plaque cavity volume (i.e., the anechoic area formed after PR) for assessing the risk of NF/SF phenomenon. In a study by Ohshima et al. [9], measurements with virtual histology intravascular ultrasound (VH-IVUS) revealed that patients with NF had significantly larger ruptured plaque cavity volumes than those with normal flow (11.5 ± 6.3 mm^3^ vs. 3.7 ± 2.2 mm^3^, *p* < 0.001). Cavity volume was independently associated with NF/SF phenomenon (OR: 1.56, 95% CI: 1.24–1.96, *p* < 0.001). Moreover, a cut-off value of ≥7.8 mm^3^ for cavity volume achieved 78.9% sensitivity and 94.1% specificity in predicting NF/SF phenomenon (area under the curve [AUC]: 0.862, 95% CI: 0.746–0.979, *p* < 0.001). This association may be explained by the pathological features of the plaque’s necrotic core. Virmani et al. [20] confirmed via postmortem analysis that ruptured plaques are characterized by larger necrotic cores, more prominent cholesterol clefts, and greater infiltration with macrophages. The necrotic core is comprised of fragile constituents such as foam cell-rich lipid deposits, intraplaque hemorrhage, and CCs. When the vulnerable culprit plaque ruptures, these substances are likely to embolize into the distal coronary artery, causing myocardial damage and subsequent NF/SF phenomenon [21,22].

For saphenous vein graft (SVG) lesions treated with PCI, IVUS-identified multiple PRs were linked to post-PCI NF/SF phenomenon [23]. Multivariate logistic regression analysis revealed that multiple PRs within the culprit SVG lesion were independent predictors of post-PCI NF/SF phenomenon (OR: 3.46, 95% CI: 1.46–8.41, *p* = 0.014).

#### 2.1.2 Attenuated Plaque

On IVUS images, attenuated plaque presents as areas of reduced echo intensity within the atherosclerotic region, with no evidence of obvious dense calcification [24] (Fig. 1B). Kawaguchi et al. [22] reported a strong association between attenuated plaque and the presence of large necrotic cores. Pathological investigations further confirmed that attenuated plaque is enriched with microcalcifications and cholesterol crystals [25,26]. These components are susceptible to mechanical fragmentation during PCI, such as balloon dilation or stent implantation. The fragmentation can subsequently trigger distal embolization, disrupting microcirculatory integrity. In a study of patients with stable coronary artery disease (CAD), Okutsu et al. [10] observed a significantly higher incidence of NF/SF phenomenon in the attenuated plaque group compared with the non-attenuated plaque group (15.7% vs. 0.2%, *p* < 0.001). The sensitivity, specificity, positive predictive value, negative predictive value, and accuracy of attenuated plaque for predicting NF/SF phenomenon were 90.9%, 88.9%, 15.7%, 99.8%, and 89.0%, respectively. A meta-analysis further validated the clinical significance of attenuated plaque [27]. Encompassing 9 studies with a total of 3833 patients, this analysis demonstrated that patients with attenuated plaque had a significantly higher incidence of TIMI 0–2 grade flow (relative risk [RR]: 4.73, 95% CI: 3.03–7.40, *p* < 0.001) following PCI, whereas the incidence of TIMI 3 grade flow was notably lower in this group (RR: 0.77, 95% CI: 0.67–0.90, *p* = 0.02).

The longitudinal length and angle of attenuated plaque are crucial for risk stratification. Endo et al. [8] demonstrated that attenuated plaque with a longitudinal length of ≥5 mm was an independent predictor of NF/SF phenomenon (OR: 20.1, 95% CI: 5.87–69.0, *p* < 0.001). In another study, Narayanan et al. [11] reported that an attenuated plaque length of ≥6.67 mm could predict NF/SF phenomenon, with a sensitivity of 69.2% and a specificity of 67.4%. The attenuation angle scoring system operates as follows: the attenuation angle is measured for each 1 mm segment of the plaque, with scores assigned on a 1–4 scale corresponding to angle ranges of <90°, 90°–180°, 180°–270°, and 270°–360°, respectively. Total scores across all segments are summed and then normalized to the analysis length to calculate an average attenuation score. Wu et al. [24] reported that an average attenuation score of ≥2 showed robust performance in predicting NF/SF phenomenon, achieving an AUC of 0.883. Furthermore, it emerged as the strongest predictor of the NF/SF phenomenon (OR: 6.586, 95% CI: 2.680–16.188, *p* < 0.001) [24].

#### 2.1.3 Positive Remodeling

Positive remodeling is defined as the condition where the ratio of the EEM cross-sectional area at the coronary lesion site to that at the reference segment exceeds 1.05 [6]. Physiologically, this phenomenon reflects the compensatory dilation of the coronary vessel wall in response to increasing plaque burden—an adaptive mechanism aimed at preserving luminal patency during the early stages of atherogenesis (Fig. 1C). Studies indicate that positively remodeled lesions frequently exhibit unstable features (e.g., thin-cap fibroatheromas), which are prone to rupture and release thrombogenic substances [28,29]. In a study of patients with acute coronary syndrome (ACS), Narayanan et al. [11] identified positive remodeling as an independent predictor of NF/SF phenomenon (OR: 6.4, 95% CI: 1.1–38.4, *p* = 0.042). Yamamoto et al. [30] further validated this association in patients with left main ACS: the remodeling index was significantly higher in the SF group (1.15 ± 0.17) than in the non-SF group (0.99 ± 0.11, *p* = 0.001), and each 0.1 increase in the remodeling index was linked to a 2.238-fold increased risk of SF (95% CI: 1.144–4.379, *p* = 0.019). The predictive value of positive remodeling is particularly notable in SVG lesions. Hong et al. [31] found that positive remodeling in SVG lesions was strongly correlated with vulnerable plaque and served as an independent predictor of post-PCI NF/SF phenomenon (OR: 2.58, 95% CI: 1.25–5.64, *p* = 0.040). This indicates the need for more rigorous preoperative assessment and meticulous intraoperative management of SVG lesions.

#### 2.1.4 Necrotic Core

VH-IVUS categorizes coronary arterial tissues into four distinct components: fibrous components, fibro-lipidic components, dense calcifications, and necrotic cores [32,33] (Fig. 1D). In a study focusing on patients with ACS, Hong et al. [26] observed notable differences in necrotic core characteristics between patients who developed NF/SF phenomenon and those with normal coronary flow following PCI. Specifically, at the minimal lumen site, the “absolute necrotic core area” and “percentage of necrotic core area relative to total plaque area” were significantly larger in the NF group compared to the normal flow group (1.6 ± 1.2 mm^2^ vs. 0.9 ± 0.8 mm^2^, *p* < 0.001; 24.5 ± 14.3% vs. 16.1 ± 10.6%, *p* = 0.001, respectively). Similarly, the “absolute necrotic core volume” and “percentage of necrotic core volume relative to total plaque volume” in the NF group were substantially higher than in the normal flow group (30 ± 24 mm^3^ vs. 16 ± 17 mm^3^, *p* = 0.001; 22 ± 11% vs. 14 ± 8%, *p* < 0.001, respectively). Multivariate regression analysis further confirmed that the percentage of necrotic core volume was the sole independent predictor of post-PCI NF/SF phenomenon in ACS patients (OR: 1.126, 95% CI: 1.045–1.214, *p* = 0.002). The underlying mechanism explaining this association may involve the structural fragility of necrotic core components. The necrotic core contains fragile substances such as lipid deposits, foam cells, intraplaque hemorrhage, and CCs. During PCI, these fragile components are prone to fragmentation and detachment. The detached debris can then form microemboli, which travel to and occlude distal microvessels, ultimately leading to the NF phenomenon [34]. Complementing these findings, a separate investigation on ACS patients by Utsunomiya et al. [12] found that the volume of necrotic plaque and percentage of necrotic plaque volume had greater predictive value for post-PCI NF/SF phenomenon compared to total plaque volume. In their study, the absolute volume of necrotic plaques and the percentage of necrotic plaques relative to total plaque volume were significantly higher in the SF group than in the normal flow group (43.3 ± 33.5 mm^3^ vs. 20.1 ± 17.2 mm^3^, *p* = 0.0004; and 19.7 ± 5.1% vs. 14.6 ± 8.3%, *p* = 0.047, respectively). Receiver Operating Characteristic (ROC) curve analysis revealed that a necrotic plaque volume threshold of ≥21.6 mm^3^ yielded the optimal predictive performance for NF/SF phenomenon, with an AUC of 0.766 (95% CI: 0.627–0.906), a sensitivity of 81.8%, and a specificity of 61.9%.

In patients with STEMI undergoing PCI, VH-IVUS revealed that the absolute necrotic core volume was significantly higher in the SF group than in the normal flow group (8.3 mm^3^ vs. 5.5 mm^3^, *p* < 0.001). Multivariate analysis further confirmed that absolute necrotic core volume was an independent predictor of postoperative NF/SF phenomenon (OR: 1.159, 95% CI: 1.030–1.305, *p* = 0.015) [35]. In a study of ACS patients, Higashikuni et al. [36] found that the percentage of necrotic core components in the culprit lesion was notably higher in patients with NF than in those with normal flow (22.1 ± 9.3% vs. 11.7 ± 7.9%, *p* = 0.0011). Even after adjusting for plaque geometric features and procedural factors, the percentage of necrotic core components remained an independent predictor of NF/SF phenomenon (OR: 1.7, 95% CI: 1.1–2.5, *p* = 0.015) [36].

In patients with SVG disease who underwent PCI following coronary artery bypass grafting (CABG), an IVUS-based study identified the absolute necrotic core area as an independent determinant of postoperative NF/SF phenomenon (OR: 2.47, 95% CI: 1.14–5.36) [37]. Furthermore, this study established a clinically meaningful threshold: an absolute necrotic core area of ≥1.1 mm^2^ conferred significant predictive value for NF/SF phenomenon following PCI in patients with SVG disease.

#### 2.1.5 Ratio of Stent Diameter to Vessel Diameter

At each lesion site, the ratio of stent diameter to vessel diameter was calculated using two key parameters: the actual vessel diameter measured via IVUS prior to stent implantation, and the pre-specified nominal stent diameter (defined at nominal inflation pressure) [13]. A study by Watanabe et al. [13] that focused on patients with STEMI identified this ratio as the only modifiable key factor for predicting post-procedural NF/SF phenomenon. Specifically, each 0.1 increase in the ratio was associated with a significant increase in SF risk (OR: 2.63, 95% CI: 1.84–3.77, *p* < 0.001). ROC curve analysis further established an optimal cut-off value of 0.71 for this ratio. Values at or above this threshold predicted NF/SF phenomenon with a sensitivity of 80.4% and a specificity of 56.9%. Additional subgroup analysis revealed that patients managed with a “moderate stent expansion strategy” (ratio <0.71) had a significantly lower incidence of NF/SF phenomenon (6.4%) compared to those treated with an “aggressive stent expansion strategy” (ratio ≥0.71, 27.1%). Notably, no significant difference was found in the incidence of ischemia-driven target vessel revascularization between the two groups, indicating that moderate stent expansion reduces SF risk without increasing the likelihood of long-term stent restenosis-related events. The predictive value of the ratio of stent diameter to vessel diameter was further validated in patients with non-ST-segment elevation myocardial infarction (NSTEMI) who underwent PCI for culprit lesions [38]. In this cohort, the ratio was significantly higher in patients who developed NF/SF phenomenon (0.77 ± 0.11) than in those with normal flow (0.71 ± 0.11, *p* = 0.03). After adjusting for potential confounding variables, multivariate logistic regression analysis confirmed the ratio was an independent determinant of SF, with each 0.1 increase in the ratio associated with a 2.06-fold higher risk (95% CI: 1.23–3.46, *p* = 0.006).

#### 2.1.6 Plaque Prolapse

**Fig. 2.**
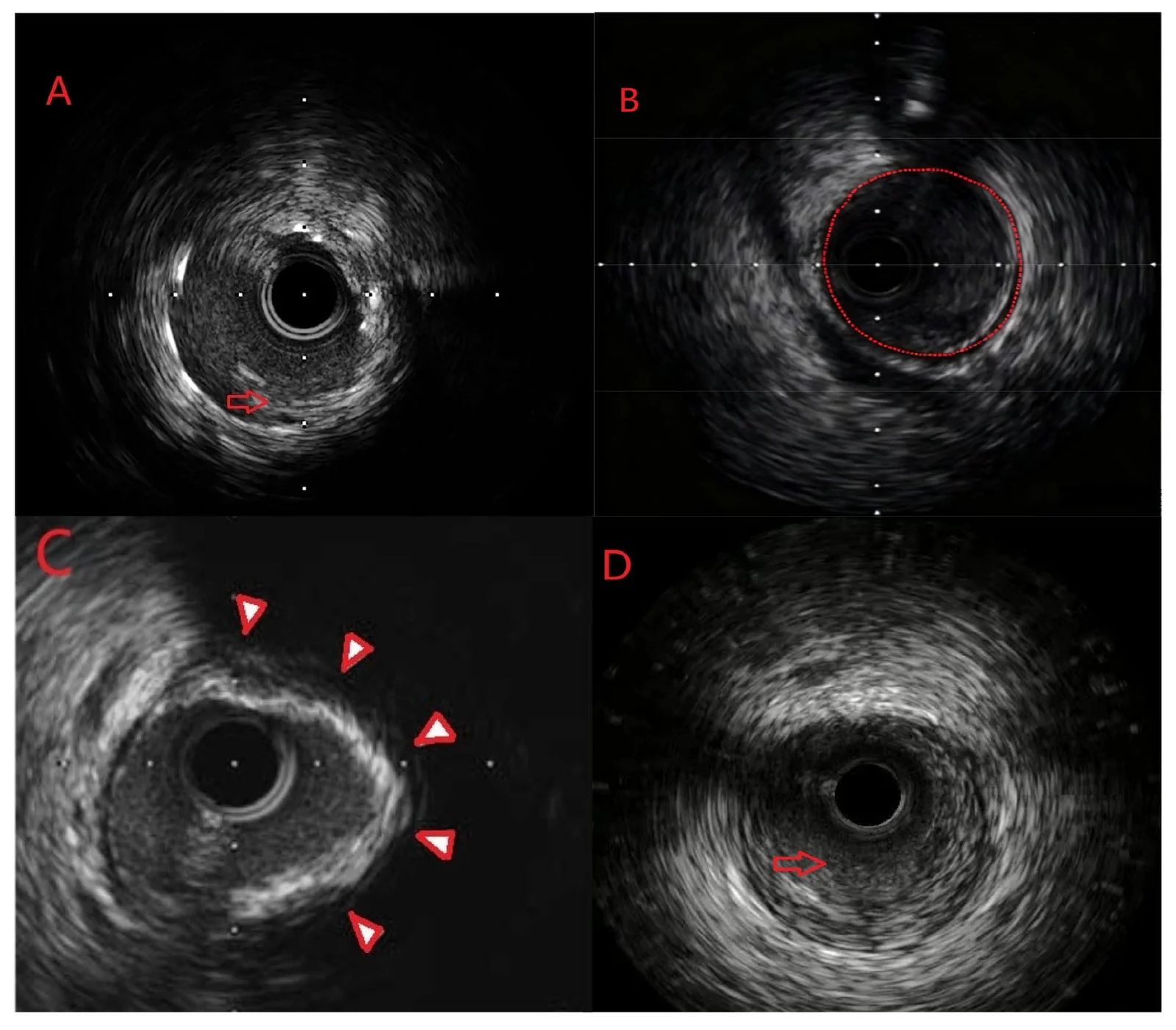
**Lesion characteristics in IVUS to predict coronary no-reflow/slow-flow phenomenon**. (A) Plaque prolapse: the protrusion of intra-stent tissue through stent struts (indicated by red arrow). (B) EEM cross-sectional area (outlined by red circle). (C) Calcification arc (indicated by red triangle). (D) Lipid plaque (indicated by red arrow). IVUS, intravascular ultrasound. EEM, external elastic membrane.

Plaque prolapse is explicitly defined as the protrusion of intra-stent tissue through stent struts. This is visualized during IVUS pullback imaging performed after stent implantation (Fig. 2A). Narayanan et al. [11] found a significant association between the length of plaque prolapse post-PCI and the development of NF/SF phenomenon. Specifically, patients with SF exhibited substantially longer plaque prolapse compared to those with normal flow (9.73 mm vs. 6.58 mm, *p* = 0.029). ROC analysis further established a clinically relevant threshold: a plaque prolapse length of >8.54 mm was correlated with post-procedural NF/SF phenomenon, with 81.8% sensitivity and 66% specificity. These authors hypothesized that penetration of extended atherosclerotic plaque attachments through stent struts may be a surrogate marker for more severe plaque embolization. This embolization subsequently disrupts microcirculatory integrity and impairs myocardial blood flow, ultimately contributing to SF. Consistent with these findings, another investigation that focused on patients with AMI post-stent implantation reported a significantly higher incidence of NF/SF phenomenon in those with tissue prolapse compared to those without (25.4% vs. 9.8%, *p* < 0.001) [39]. Multivariate logistic regression analysis further confirmed tissue prolapse as an independent predictor of the composite endpoint, encompassing acute in-stent thrombosis and NF (OR: 2.551, 95% CI: 1.315–4.952, *p* = 0.006).

#### 2.1.7 EEM Cross-Sectional Area

The EEM cross-sectional area is defined as the anatomical region bounded by the interface between the ultrasound medium and the arterial adventitia. It is quantified by delineating two key structural boundaries: the leading edge of the arterial adventitia and the cross-sectional area of the vascular lumen [6] (Fig. 2B). In a study specifically focused on patients with AMI, Tanaka et al. [40] found that the EEM cross-sectional area of culprit lesions was significantly larger in patients who developed post-reperfusion NF/SF phenomenon compared to those with normal coronary flow [40]. Furthermore, multivariate logistic regression analysis confirmed that the EEM cross-sectional area of the culprit lesion was an independent predictor of post-reperfusion NF/SF phenomenon in AMI patients (OR: 1.55, 95% CI: 1.01–2.38, *p* < 0.05). These findings support the hypothesis that lesions located in larger-caliber coronary vessels confer a higher risk of NF. A plausible mechanistic explanation is that larger vessels have a greater anatomical capacity to accommodate larger volumes of atherosclerotic plaque or thrombus. Upon intervention of these lesions (e.g., during PCI), the increased burden of plaque/thrombus elevates the likelihood of distal embolization of fragmented material—an important contributor to MVO and subsequent NF. The association between larger vessel size and NF/SF phenomenon risk was further validated in a study by Kimura et al. [41]. After adjusting for potential confounding variables (e.g., patient demographics, comorbidities, and procedural factors), these researchers found that a larger reference lumen area (specifically, >15.3 mm^2^) was significantly and independently associated with post-procedural NF/SF phenomenon (OR: 3.08, 95% CI: 1.40–6.76, *p* = 0.005).

#### 2.1.8 Vasa Vasorum

Coronary vasa vasorum (VV) is a network of microvessels embedded within the coronary arterial wall. VV are frequently observed in patients with AMI, thin-cap fibroatheroma (TCFA) lesions, or PR [42,43]. Clinically, VV are recognized as reliable morphological markers for identifying vulnerable plaques and tracking plaque progression [44,45]. On IVUS images, they present as small (<1 mm), tubular or cystic hypoechoic structures, typically localized within ≤1 mm of the outer media [46]. In a study of patients with ACS conducted by Wu et al. [47], lesions associated with VV exhibited distinct pathological features at the minimal lumen area, including larger necrotic cores and fibrous areas, smaller dense calcification areas, greater overall plaque volume, and a higher prevalence of TCFAs and attenuated plaques. Multivariate logistic regression analysis further confirmed that both VV and plaque volume were independent predictors of post-stent implantation NF/SF phenomenon (VV: OR: 5.13, 95% CI: 1.19–15.32, *p* = 0.002; plaque volume: OR: 4.79, 95% CI: 1.08–9.01, *p* = 0.005). A plausible mechanistic explanation for this association lies in the procedural complications induced by VV-related plaque vulnerability, as during PCI, balloon dilation may trigger intraplaque hemorrhage and rupture of TCFAs. These events release fragmented plaque debris, which then embolizes to distal microvessels and ultimately induces the NF/SF phenomenon.

#### 2.1.9 Number of Reverberations and Large Calcification Arc at the Minimal Lumen Area (MLA)

Microemboli originating from calcified lesions have been identified as the primary cause of NF/SF phenomenon following rotational atherectomy (RA) [48]. In IVUS imaging, reverberations are defined as arc-shaped, hyperechoic lines that appear posterior to calcified regions, typically presenting as single or multiple equidistant structures [6]. Jinnouchi et al. [49] found that the maximum number of reverberations and a larger calcification arc at the MLA may predict NF/SF phenomenon after RA, with statistically significant associations observed between these parameters and NF/SF phenomenon. For each additional reverberation, the odds of NF/SF phenomenon increased by 1.49-fold (OR: 1.49, 95% CI: 1.07–2.07, *p* = 0.02). Near-circular calcification (≥300°) at the MLA was independently associated with a 2.21-fold higher risk of NF/SF phenomenon (OR: 2.21, 95% CI: 1.13–4.32, *p* = 0.02). Notably, the incidence of NF/SF phenomenon showed a clear positive correlation with the maximum number of reverberations: 2.2% (n = 0), 11.9% (n = 1), 19.5% (n = 2), 22.5% (n = 3), and 44.4% (n = 4). The underlying mechanism linking these parameters to the NF/SF phenomenon involves two key pathways. Firstly, a larger calcification arc at the MLA increases vascular resistance, impeding the effective penetration of RA devices through the lesion. Secondly, complex lesions with extensive calcification (larger arc) often require prolonged RA procedural time, thereby increasing the generation of calcified microemboli. These microemboli then occlude distal microvessels, ultimately increasing the risk of NF/SF phenomenon [49] (Fig. 2C).

#### 2.1.10 Stent Overexpansion

Stent expansion is quantified using the following formula: stent expansion = intra-stent MLA / mean reference lumen cross-sectional area (CSA). The mean reference lumen CSA is calculated as: (proximal reference lumen CSA + distal reference lumen CSA) / 2. Clinically, stent expansion is categorized into three grades based on this ratio: under-expansion (<80%), optimal expansion (80–100%), and over-expansion (>100%). In a study of patients with STEMI undergoing their first PCI, Li et al. [50] reported two key findings: the overexpansion group exhibited a significantly higher volume and proportion of necrotic plaques compared to other groups; and stent expansion was negatively associated with blood flow velocity, with a reduction of −0.14 cm/s for each 1% increase in the expansion ratio (*p* < 0.01). After adjusting for confounding factors related to overexpansion, multivariate analysis further confirmed that stent overexpansion (>100%) was an independent risk factor for post-PCI NF/SF phenomenon (hazard ratio [HR]: 1.27, 95% CI: 1.20–2.52, *p* = 0.02). The underlying mechanism linking stent overexpansion to deteriorated vascular function and poor prognosis in STEMI patients may be explained by the following pathological process. Lesions prone to overexpansion often harbor a higher prevalence of unstable pathological features, including vulnerable plaques, large lipid pools, expanded necrotic cores, and adherent thrombi. During PCI (e.g., stent implantation or post-implantation balloon dilation), these unstable components are more likely to rupture and extrude through the widened stent struts [40]. The extruded debris then forms microemboli, which occlude distal microvessels and increase microcirculatory obstructive resistance, thus directly contributing to the NF/SF phenomenon.

#### 2.1.11 Estimated Lipid Volume

Clinically, the estimated lipid volume (lipid area at the MLD site × total stent length)—measured via a simple and rapid assessment prior to PCI—serves as a reliable metric for identifying lesions at high risk of post-PCI NF/SF phenomenon [14]. Research has established a critical threshold for this parameter: an estimated lipid volume of 132.6 mm^3^ optimally predicts NF/SF phenomenon, with an AUC of 0.719. Notably, this predictive performance is comparable to that of actual lipid volume. Lesions with an estimated lipid volume ≥132.6 mm^3^ were associated with a significantly higher risk of NF/SF phenomenon during elective stent implantation (OR: 4.35, 95% CI: 1.67–12.7, *p* = 0.0024) [14]. Complementary evidence from a separate study highlights the predictive value of coronary lipid volume percentage (% LV), defined as the ratio of lipid volume to total plaque volume and quantified via IVUS [15]. Patients who developed NF/SF phenomenon exhibited a significantly higher % LV than those with normal reflow [15]. ROC curve analysis identified a % LV cut-off of 62% for the prediction of NF/SF phenomenon, with a sensitivity of 48%, specificity of 81%, and AUC of 0.67. The positive predictive value for % LV >62% was 33%, and the negative predictive value was 87%. Multivariate logistic regression analysis further confirmed that % LV was an independent predictor of NF/SF phenomenon (HR: 4.5, 95% CI: 1.6–13.4, *p* < 0.01) (Fig. 2D). The AUC of IVUS-derived % LV was 0.67, reflecting only moderate predictive ability. A critical interpretation of this data is that while an AUC of 0.67 indicates a certain degree of association between IVUS-derived % LV and the target clinical outcome, it falls well short of the high predictive performance (e.g., AUC ≥0.8) required for a reliable standalone clinical tool. This moderate AUC value highlights the inherent limitations of relying on a single predictive indicator, while also casting doubt on the practical value of IVUS-derived % LV as an independent clinical tool for risk stratification or outcome prediction. Single indicators, by their very nature, capture only a limited aspect of the complex pathophysiological processes underlying cardiovascular diseases. Indeed, factors such as plaque morphology, inflammatory status, hemodynamic changes, and patient-specific clinical characteristics (e.g., age, comorbidities, lifestyle) are all integral to accurate prediction, but are not accounted for by % LV alone. Consequently, the moderate predictive ability suggested by this AUC value underscores the imperative of developing multi-modal or multi-parameter prediction models. The integration of IVUS-derived % LV with other complementary indicators (e.g., coronary computed tomography angiography findings, serum biomarkers, imaging-derived plaque features) could synergistically enhance predictive accuracy, overcome the limitations of single indicators, and provide more robust and clinically actionable information for decision-making. This issue will be discussed further in the future perspectives section.

### 2.2 Optical Coherence Tomography (OCT)

OCT operates on the principle of near-infrared light interference, boasting an ultra-high resolution of 10–20 μm. It is currently the most advanced intravascular imaging modality with the highest resolution available in clinical practice. OCT enables clear and detailed visualization of critical vascular microstructures, including precise measurement of fibrous cap thickness, accurate identification of macrophage infiltration, and clear delineation of CCs [51]. The core advantage of OCT lies in its exceptional ability to characterize plaque surface features and detect subtle micro-lesions with high precision, making it particularly valuable for assessing plaque stability in patients with ACS. However, a limitation of OCT is that its penetration depth is relatively limited (only 1–2 mm), thus preventing effective visualization of deep vascular structures such as the EEM. This constraint represents a major drawback in scenarios requiring comprehensive evaluation of vascular wall anatomy [52]. Through extensive clinical research, OCT has so far been shown to identify several key imaging indicators that hold predictive value for the occurrence of the coronary NF/SF phenomenon, as detailed below (Table 2, Ref. [53,54,55]).

**Table 2.** **Summarized table of OCT in predicting coronary no-reflow/slow-flow phenomenon**.

Marker	Study	Design	Population	Sample size	Cut-off	Sensitivity	Specificity	AUC	OR	95% CI	*p*	Advantages	Limitations
fibrous cap thickness	Gui SJ et al. [53]	RSC	AMI	196	<55 μm	0.935	0.967	0.985	0.874	0.814–0.940	<0.001	High resolution, detailed plaque characterization	Ostial disease, very large vessels, require blood displacement
length of thin-cap fibroatheromas	Gui SJ et al. [53]	RSC	AMI	196	≥9.5 mm	0.804	0.873	0.875	3.356	0.800–0.949	<0.001		
thin-cap fibroatheroma	Gamou T et al. [54]	RSC	ACS and CCS	126	NP	NP	NP	NP	NP	3.02–50.31	0.0005		
cholesterol crystals	Katayama Y et al. [55]	RSC	STEMI	182	12	52%	82%	0.678	1.06	0.562–0.776	0.001		
lipid arc	Katayama Y et al. [55]	RSC	STEMI	183	139°	71%	67%	0.688	1.01	0.575–0.782	<0.001		

ACS, acute coronary syndrome; AMI, acute myocardial infarction; AUC, area under the curve; CCS, chronic coronary syndromes; CI, confidence interval; NP, not reported; OCT, optical coherence tomography; OR, odds ratio; RSC, retrospective single center; STEMI, ST-segment elevation myocardial infarction.

#### 2.2.1 Thin-Cap Fibroatheroma (TCFA)

**Fig. 3.**
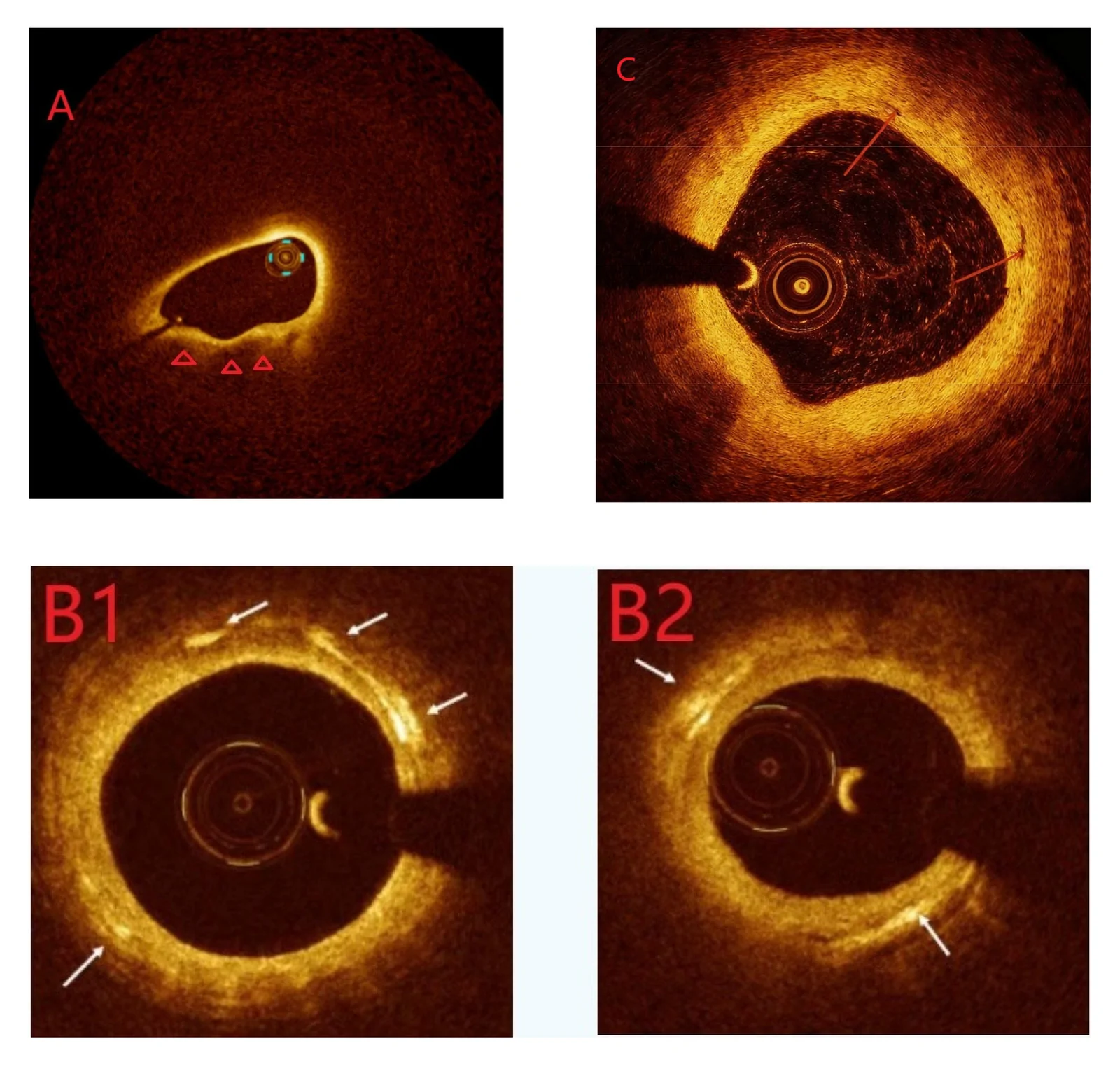
**Lesion characteristics in OCT to predict coronary no-reflow/slow-flow phenomenon**. (A) Thin-cap fibroatheroma (indicated by red triangle). (B) Cholesterol crystals: thin, high-signal linear structures with distinct boundaries (B1 and B2, indicated by white arrows). (C) Vasa vasorum: specific subtype of these microvessels that extend from the arterial adventitia into the plaque parenchyma (indicated by red arrows). OCT, optical coherence tomography.

Fibrous cap thickness is a core indicator for evaluating plaque stability, and OCT enables quantitative measurement of the fibrous cap with an exceptional precision of 10 μm. Extensive research has consistently demonstrated that a thinner fibrous cap correlates with an elevated risk of PR, which in turn increases the likelihood of developing the NF/SF phenomenon [53] (Fig. 3A). When the fibrous cap thickness was <55 μm, the AUC for predicting NF/SF phenomenon reached an impressive 0.985 (95% CI: 0.968–1.000, *p* < 0.001), accompanied by a sensitivity of 0.935 and specificity of 0.967 [53]. Beyond its thickness, the length of TCFAs also holds clinical significance. A TCFA length of ≥9.5 mm exhibited notable predictive value for NF/SF phenomenon, with an AUC of 0.875 (95% CI: 0.800–0.949, *p* < 0.001), a sensitivity of 0.804, and a specificity of 0.873. A subsequent study by Gamou et al. [54] provided additional compelling evidence, confirming that TCFAs are robust predictors of deteriorated coronary flow following PCI in patients with either ACS or chronic coronary syndrome (CCS) (HR: 12.32, 95% CI: 3.02–50.31, *p* = 0.0005). The underlying mechanism driving this association lies in the inherent vulnerability of TCFAs. These lesions are highly prone to rupture, releasing thrombogenic components into the coronary circulation that trigger a cascade of events (e.g., microembolization, MVO). Ultimately, this leads to impaired coronary flow, which manifests as the NF/SF phenomenon.

#### 2.2.2 Cholesterol Crystals (CCs) and Lipid Arc

On OCT images, CCs manifest as thin, high-signal linear structures with distinct boundaries. This key morphological feature enables clear differentiation from the boundary-less, high-signal regions corresponding to macrophages [56,57] (Fig. 3B1, Fig. 3B2). A landmark study identified both the number of CCs and lipid arc as independent positive predictors of NF/SF phenomenon [55]. Specifically, a CC count of ≥12 emerged as an independent predictor of NF/SF phenomenon, with an AUC of 0.678 (95% CI: 0.562–0.776, *p* = 0.001). Similarly, a lipid arc of ≥139° showed significant predictive efficacy for NF, achieving an AUC of 0.688 (95% CI: 0.575–0.782, *p* < 0.001). Notably, the combined application of these two metrics substantially enhanced their predictive performance. When both the CC count (≥12) and lipid arc (≥139°) reached their respective thresholds, the prediction of NF/SF phenomenon increased to 93% specificity and 86% accuracy. This integrated approach provides a more precise and clinically actionable tool for identifying high-risk lesions, thereby guiding targeted interventional strategies. The presence of CCs is pathologically indicative of two critical plaque characteristics: a highly mature necrotic core and compromised mechanical stability. Such plaques are inherently vulnerable to fragmentation during PCI, which in turn predisposes to microvascular embolization, a key pathological step in the development of the NF/SF phenomenon. The prognostic significance of lipid arc was further validated in a patient cohort with non-ST-segment elevation acute coronary syndrome (NSTE-ACS). Multivariate regression analysis revealed a dose-dependent relationship between lipid arc and NF risk, with each 1° increase in lipid arc associated with a 1.018-fold higher risk of NF/SF phenomenon (95% CI: 1.004–1.033, *p* = 0.01) [58]. This finding highlights that lipid-rich plaque components represent a shared risk factor for microcirculatory injury across different subtypes of ACS, underscoring the universal relevance of lipid arc assessment in ACS patients undergoing PCI.

#### 2.2.3 Vasa Vasorum (VV)

VV serves as the primary source of neovascularization within atherosclerotic plaques. OCT enables the clear identification of internally penetrating VV—a specific subtype of these microvessels that extend from the arterial adventitia into the plaque parenchyma (Fig. 3C). Clinical evidence underscores the prognostic relevance of this VV subtype to post- PCI flow disturbances, with a pivotal study demonstrating that lesions harboring internally penetrating VV were associated with a significantly higher incidence of NF/SF phenomenon after PCI compared to those without such vessels (42% vs. 13%, *p* = 0.007). Multivariate analysis further confirmed internally penetrating VV as an independent predictor of NF/SF phenomenon (OR: 4.23, 95% CI: 1.05–17.01, *p* = 0.042) [59]. The mechanistic link between internally penetrating VV and the impaired NF/SF phenomenon lies in their close association with plaque inflammation and instability. These microvessels exhibit structural fragility, rendering them prone to leakage or rupture—events that elevate the risk of intraplaque hemorrhage. By disrupting plaque integrity and promoting the release of prothrombotic or proinflammatory mediators, intraplaque hemorrhage indirectly precipitates microcirculatory obstruction, thereby contributing to the development of both NF/SF phenomenon.

### 2.3 Near-Infrared Spectroscopy (NIRS)

By leveraging the differential absorption of near-infrared light by various biological substances, NIRS enables quantitative assessment of the lipid core burden within coronary atherosclerotic plaques. Its core diagnostic metric is the maximum 4-mm lipid core burden index (maxLCBI_4mm_), which is defined as the highest lipid core burden value measured across any continuous 4-mm segment along the longitudinal axis of the target vessel [60,61,62,63]. A key advantage of NIRS lies in its unique ability to directly quantify lipid components within plaques, independent of the structural imaging modality. This distinctive feature renders it particularly valuable for identifying “occult” lipid-rich plaques—lesions that may lack overt structural abnormalities on traditional imaging, but which harbor significant lipid accumulation, a critical hallmark of plaque vulnerability. However, NIRS has a notable limitation in that it cannot provide information regarding vascular structural characteristics, such as lumen geometry, plaque morphology, or vessel wall integrity. Consequently, to achieve a comprehensive “composition + structure” assessment of coronary lesions, NIRS must be used in combination with IVUS or OCT, which complement its functional strengths with detailed anatomical visualization [64] (Table 3, Ref. [65,66,67]).

**Table 3.** **Summarized table of NIRS in predicting coronary no-reflow/slow-flow phenomenon**.

Marker	Study	Design	Population	Sample size	Cut-off	Sensitivity	Specificity	AUC	OR	95% CI	*p*	Advantages	Limitations
MaxLCBI_4mm_	Suzuki N et al. [65]	RSC	ACS	115	578	100%	64.4%	0.849	NP	NP	NP	Detects lipid core plaque, adjunct to IVUS or OCT	Limited indication as standalone modality
	Suzuki N et al. [65]	RSC	CCS	64	480	100%	63.6%	0.851	NP	NP	NP		
	Ozaki Y et al. [67]	RSC	ACS	371	719	69.8%	72.1%	NP	1.008	1.005–1.012	<0.001		
	Hosoda H et al. [66]	RSC	Large calcified lesions (maximum calcification arc ≥180°)	189	679	64.3%	86.3%	0.76	1.60	1.32–1.94	<0.001		

ACS, acute coronary syndrome; AUC, area under the curve; CCS, chronic coronary syndromes; CI, confidence interval; MaxLCBI_4mm_, maximum 4-mm lipid core burden index; NIRS, near-infrared spectroscopy; NP, not reported; OR, odds ratio; RSC, retrospective single center.

Numerous studies have established a robust positive correlation between the maxLCBI_4mm _and the risk of developing NF/SF phenomenon. A pivotal study of patients with either ACS or CCS demonstrated that maxLCBI_4mm _values were significantly elevated in the SF group compared to the normal flow group (740 ± 147 vs. 471 ± 223, *p* < 0.001) [65]. Notably, when applying modality-specific thresholds, the sensitivity for predicting SF reached 100%. This was observed in ACS patients with a maxLCBI_4mm_ of ≥578, and in CCS patients with a maxLCBI_4mm _of ≥480. Further validation came from a study by Terada et al. [68], which utilized cardiac magnetic resonance imaging to confirm that a maxLCBI_4mm_ of >600 was the optimal threshold for predicting MVO following PCI in patients with STEMI. This finding reinforces the close association between elevated maxLCBI_4mm_ and microcirculatory injury, a key pathological driver of NF and SF. In a separate analysis focusing exclusively on ACS patients [67], the incidence of NF was substantially higher in those with a maxLCBI_4mm _of ≥400 compared to those with a maxLCBI_4mm_ of <400 (17.5% vs. 2.5%, *p* < 0.001). Multivariate regression analysis further confirmed that maxLCBI_4mm _was an independent predictor of NF (OR: 1.008, 95% CI: 1.005–1.012, *p* < 0.001). These findings underscore the clinical utility of maxLCBI_4mm_ as a reliable, quantitative marker for stratifying NF risk in the ACS population.

The predictive utility of maxLCBI_4mm _is particularly pronounced with calcified lesions. Calcified plaques have long been regarded as relatively stable in traditional clinical perspectives. However, a seminal study by Hosoda et al. [66] revealed the critical insight that lipid components within calcified lesions substantially elevate the risk of NF. Specifically, in lesions with small calcifications (defined by a maximum calcification arc <180°), a maxLCBI_4mm _threshold of ≥585 demonstrated robust predictive performance for NF, achieving an AUC of 0.72 (*p* < 0.001). In contrast, for lesions with large calcifications (maximum calcification arc ≥180°), the optimal maxLCBI_4mm _threshold for the prediction of NF was higher, at 679, with an even more favorable AUC of 0.76 (*p* = 0.001). Furthermore, multivariate analysis confirmed that maxLCBI_4mm _in large calcified lesions was as an independent predictor of NF (OR: 1.60, 95% CI: 1.32–1.94, *p* < 0.001). This finding directly challenges the conventional notion of “stable calcified plaques”, and emphasizes the clinical imperative of assessing the lipid component burden within calcified lesions. Notably, even calcified plaques with significant lipid accumulation pose a tangible risk of PCI NF, requiring targeted preoperative evaluation and procedural planning.

Beyond its utility in predicting NF, NIRS also has value in predicting the post-PCI TIMI flow grade, a key indicator of coronary hemodynamic recovery. A clinical study of 423 patients provided compelling evidence for this predictive ability [69]. Specifically, high LCBI (≥354) was identified as an independent factor associated with reduced TIMI flow (i.e., TIMI 0–2 grade) (OR: 2.59, 95% CI: 1.33–5.04, *p* = 0.005). When focusing on the maxLCBI_4mm_, a threshold of ≥354 showed predictive efficacy for reduced TIMI flow, achieving an AUC of 0.61 (95% CI: 0.54–0.67, *p* = 0.001). These findings highlight two critical insights: first, the influence of plaque lipid components on post-interventional coronary flow is broader than previously recognized—extending beyond the binary distinction of “NF vs. reflow” to also impact the rate of flow recovery. Second, maxLCBI_4mm _emerges as a clinically actionable reference indicator for intra-procedural flow assessment. By integrating this lipid-focused metric into real-time decision-making during PCI, clinicians can more proactively identify patients at risk of suboptimal flow recovery and tailor interventional strategies (e.g., adjunctive anti-embolic measures or modified stent expansion protocols) accordingly to mitigate such risks.

## 3. Discussion

Several studies have performed head-to-head comparisons of IVUS and OCT for PCI guidance. In the OPINION Trial, the use of both imaging techniques led to similar 1-year clinical outcomes, with very low rates of 8-month angiographic binary in-segment restenosis (6.2% and 6.0%) [70]. In addition, the rate of 12-month target vessel failure (defined as a composite of cardiac death, target-vessel related myocardial infarction, and ischaemia-driven target vessel revascularization) was 5.2% and 4.9% for OCT and IVUS, respectively. However, it remains unknown whether the additional procedures performed on the basis of OCT or IVUS evaluation reduce the incidence of target vessel failure in relatively simple lesions. Moreover, this trial was not sufficiently powered to detect clinically relevant outcomes, and did not test a broad range of patients with high-risk lesion subsets. The OCTIVUS trial reported that OCT-guided PCI was noninferior to IVUS-guided PCI with respect to the incidence of a composite of death from cardiac causes, target vessel–related myocardial infarction, or ischemia-driven target vessel failure at 1 year [71]. This trial provided relevant clinical evidence on the relative efficacy and safety of OCT- and IVUS-guided PCI in a large and potentially representative population in routine practice. The RENOVATE-COMPLEX-PCI trial demonstrated that either IVUS- or OCT-guided PCI significantly reduced MACE compared to angiography-guided PCI in patients with complex coronary artery lesions [72]. Over a 2.1-year follow-up period, the imaging-guided group had a lower incidence of cardiac death, target-vessel myocardial infarction, and target-vessel revascularization (7.7% vs. 12.3%, respectively; HR: 0.64; *p* = 0.008). Several recent meta-analyses and observational studies have reported that both IVUS and OCT outperform angiography alone in PCI guidance by reducing stent-related complications and improving procedural outcomes [73,74,75,76]. Although OCT may be superior for procedural optimization (stent expansion, detection of malapposition), the current evidence for reducing long-term adverse events (e.g., stent thrombosis, restenosis) may be stronger for IVUS. To date, there have been no studies utilizing NIRS for the optimization of PCI.

The optimal imaging strategy for different clinical contexts of coronary atherosclerotic heart disease should be selected individually according to pathological and physiological characteristics, and to the diagnostic and therapeutic goals. Specifically, emergency coronary angiography combined with primary PCI is the core strategy for STEMI, with intravascular imaging (IVUS/OCT) only used as a supplementary intraoperative tool for complex cases [77]. For ACS patients, OCT is the preferred method to accurately identify culprit lesion mechanisms (e.g., plaque erosion, rupture, or calcified nodules) [77], guiding individualized management such as the avoidance of stent implantation in plaque erosion [78]. In stable CAD, IVUS optimally assesses vascular structure, plaque burden, and intervention criteria [79]. For calcified lesions, OCT uniquely affords the ability to measure and characterize the thickness of calcium in heavily calcified stenoses [80], since light (as opposed to ultrasound) is not attenuated by calcium. Superficial calcium requires the measurement of OCT thickness to guide rotational atherectomy/shockwave lithotripsy [81]. IVUS, OCT, and NIRS can all identify and evaluate lipid-rich plaques. Currently, there are no head-to-head studies comparing the efficacy of intravascular imaging in predicting the prognosis of lipid-rich plaques. In clinical practice, the most appropriate imaging method can be selected to evaluate lipid-rich plaques based on the advantages and disadvantages of different intravascular imaging techniques.

It has been established that MVO is the core pathophysiological mediator of the NF/SF phenomenon. The essence of MVO is structural destruction or dysfunction of the coronary microvascular bed, leading to impaired myocardial perfusion and further exacerbation of myocardial ischemia and hypoxia [82]. In recent years, advances in imaging technologies such as OCT, IVUS and NIRS have led to the identification of several high-risk imaging features, including PR, TCFA, lipid pool, positive remodeling, and macrophage accumulation. These features not only represent the core hallmarks of atherosclerotic plaque vulnerability, but also directly mediate or participate in the initiation and progression of MVO through multiple specific pathophysiological pathways. The close correlation between these features provides an important theoretical basis and practical foundation for the early clinical identification of MVO risk and optimization of intervention strategies.

PR is the most common high-risk imaging feature in patients with ACS [16]. Its association with MVO is primarily mediated through distal embolization of atherothrombotic debris, accompanied by induced thrombosis that further aggravates obstruction [83]. The central pathological change of PR is disruption of fibrous cap integrity, resulting in the release of lipid core, necrotic tissue, inflammatory cells, and other atheromatous debris into the bloodstream [29]. These tiny debris can drift distally along the coronary flow and cause mechanical obstruction within the coronary microvasculature (generally <200 μm in diameter), leading to distal embolic MVO. This is one of the critical triggers of the NF/SF phenomenon after PCI [83]. At the pathophysiological level, in addition to debris embolization, PR can expose plaque components and activate the coagulation cascade, thereby promoting platelet aggregation and thrombus formation. The thrombus fragments can further occlude microvessels [83]. Meanwhile, the inflammatory response at the rupture site releases abundant inflammatory mediators, aggravating microvascular endothelial injury, reducing microvascular compliance, and indirectly contributing to the development of MVO [84].

As an important precursor lesion of PR, TCFA serves as a high-risk predictor of MVO [85]. The mechanistic link between TCFA and MVO involves both the amplified effect of atheromatous debris embolization and direct microvascular injury induced by lipid components [84,86]. The key imaging characteristics of TCFA include a fibrous cap thickness <65 μm, and a large lipid core relative to the total plaque volume [29]. Such plaques possess a fragile fibrous cap that is highly susceptible to rupture under hemodynamic stress or inflammatory stimulation, and their lipid-rich cores provide sufficient material for debris embolization. Compared with conventional PR, TCFA rupture releases smaller and more abundant lipid debris, which are prone to occluding distal microvessels. Moreover, these lipid constituents can induce microvascular endothelial cell injury, inhibit the activity of endothelial nitric oxide synthase (eNOS), reduce nitric oxide (NO) release, cause abnormal microvascular vasoconstriction, and further exacerbate MVO [84].

The size and stability of the lipid pool directly influence the risk of MVO. A larger lipid pool and poorer plaque stability result in greater release of debris upon rupture. Furthermore, CCs within the lipid pool can stimulate intraplaque inflammation, promote macrophage accumulation, and induce the release of inflammatory mediators. This indirectly exacerbates microvascular spasm and endothelial injury, thereby establishing a vicious cycle of: lipid pool expansion → inflammatory activation → plaque rupture → debris embolization → MVO [29].

Positive remodeling is characterized by high plaque burden with relatively mild luminal stenosis and increased external vessel diameter, representing a key imaging marker of vulnerable plaques. Its association with MVO is mainly mediated by hemodynamic abnormalities that increase the risk of microvascular spasm and embolization [29]. Plaques with positive remodeling are mostly non‑calcified soft plaques with a rich lipid content and relatively preserved vascular wall elasticity. Compensatory vascular expansion leads to uneven wall stress distribution and disturbed blood flow with local turbulence. Such abnormal hemodynamics directly stimulate microvascular endothelial cells, trigger microvascular spasm, reduce luminal diameter and myocardial perfusion, and consequently promote MVO. Meanwhile, positive remodeling is frequently accompanied by increased plaque instability, predisposing to rupture or surface erosion and the release of atheromatous debris. Disturbed flow further facilitates distal migration of debris and raises the risk of microembolization [87,88,89]. In addition, positive remodeling is associated with enhanced vascular inflammation and elevated matrix metalloproteinase (MMP) activity, which contribute to structural deterioration of the vessel wall, reduced microvascular compliance, and increased sensitivity to vasoconstrictive stimuli, further aggravating microvascular dysfunction [90].

Macrophage accumulation is a central imaging and pathological feature of plaque inflammatory activation. It acts as a critical bridge linking plaque vulnerability to MVO, mainly via inflammation‑mediated microvascular endothelial injury, spasm, and inflammatory edema [90]. As the predominant inflammatory cells within plaques, macrophages ingest lipids to form foam cells and accumulate at the interface between the lipid core and fibrous cap, secreting multiple proinflammatory mediators, including tumor necrosis factor‑α (TNF‑α) and interleukin‑1 (IL‑1). These mediators directly injure microvascular endothelial cells, disrupt endothelial barrier integrity, increase microvascular permeability, and trigger inflammatory edema that compresses microvessels and impairs perfusion [91]. Inflammatory mediators also induce contraction of vascular smooth muscle cells, provoke microvascular spasm, and activate the coagulation cascade to enhance platelet aggregation and microthrombosis, further worsening MVO. Meanwhile, MMPs secreted by macrophages accelerate fibrous cap degradation and promote PR, indirectly increasing the risk of debris embolization and thus creating a pathological cascade of: macrophage accumulation → inflammatory activation → plaque instability → MVO [29,90].

These high‑risk imaging features do not exist in isolation but interact and synergize, forming a complex regulatory network that collectively contributes to the pathophysiology of MVO. For instance, plaques with positive remodeling typically contain large lipid pools and often progress to TCFA, while TCFA formation is accompanied by extensive macrophage accumulation that amplifies inflammation, further weakening fibrous cap stability and ultimately precipitating PR. The released debris causes distal embolization and simultaneously triggers microvascular spasm and ischemia–reperfusion injury, with the multiple pathways converging to induce and aggravate MVO. As a major pathophysiological mechanism of MVO, ischemia–reperfusion injury is closely related to all of the above high‑risk imaging characteristics. Vascular occlusion caused by plaque rupture, TCFA, and other lesions leads to myocardial ischemia, while the abrupt restoration of flow following revascularization, such as PCI, triggers oxidative stress and inflammatory responses with excessive production of reactive oxygen species (ROS). This further damages the microvascular endothelium, increases permeability, induces edema and leukocyte infiltration, and worsens MVO. The self‑reinforcing cycle of ischemia → reperfusion → inflammatory edema → MVO acts synergistically with macrophage‑driven inflammation to amplify microvascular injury [92].

Clarifying the links between high‑risk imaging features and the pathophysiological pathways of MVO has substantial practical value from a clinical perspective. First, the identification of PR, TCFA, enlarged lipid pool, positive remodeling, macrophage accumulation, and other features using imaging modalities enables early risk stratification for MVO, providing a rationale for risk assessment in ACS patients. For patients with multiple high‑risk features, vigilance for MVO is warranted and prophylactic interventions should be considered [93]. Second, understanding these mechanistic connections allows the use of optimized therapeutic strategies: for debris embolization, intensified antiplatelet and anticoagulant therapies can reduce thrombosis and debris release; for inflammation‑mediated endothelial injury and spasm, anti‑inflammatory agents and endothelial protective drugs may suppress inflammation and microvascular constriction; for large lipid pools and TCFA, intensive lipid‑lowering therapy can reduce lipid deposition, increase fibrous cap thickness, stabilize plaques, and lower MVO risk at the source [94].

## 4. Clinical Perspectives

The ultimate goal of predicting coronary NF and SF phenomena using intravascular imaging is to translate predictive insights into actionable clinical strategies that improve patient outcomes. When clinicians identify NF/SF risk factors via intravascular imaging techniques—such as OCT, IVUS, or NIRS—the procedural approach, administration of medication, pre-stent preparation of occluded vessels, and selection of stents/balloons are comprehensively optimized to mitigate risk and enhance myocardial perfusion.

In terms of adjusting the procedural approach, intravascular imaging enables clinicians to move from a “one-size-fits-all” strategy to personalized intervention. For example, the high resolution of OCT facilitates identification of TCFAs, PR with thrombosis, and lipid-rich plaques—all of which are key risk factors for NF [85]. Additionally, IVUS or OCT can indirectly guide the assessment of microvascular function. When imaging indicates a high risk of MVO, clinicians may adopt a stepwise revascularization strategy instead of immediate stenting, thereby reducing the burden of ischemia-reperfusion injury (IRI) on the microcirculation [82].

The administration of medication can be significantly tailored based on the NF/SF risk factors detected by intravascular imaging. For patients identified as having a high thrombus burden (e.g., by OCT or IVUS) or with vulnerable plaques, intensified antiplatelet and anticoagulant therapy is recommended to reduce thromboembolic risk. Furthermore, intracoronary administration of vasodilators (e.g., nitroglycerin, verapamil) or anti-inflammatory agents (e.g., adenosine) is proactively used in high-risk patients, as imaging-derived risk stratification helps to identify those most likely to benefit from targeted microcirculatory protection [95,96]. Notably, patients with imaging-detected endothelial dysfunction (e.g., irregular vessel walls, reduced vasoreactivity) may benefit from pre-PCI statin loading, as statins improve endothelial function and reduce IRI-related microvascular damage [34,96].

Pre-stent preparation of occluded vessels can be optimized under the guidance of intravascular imaging to minimize NF/SF risk. For occluded vessels with imaging-detected heavy calcification, severe plaque burden, or thrombus, clinicians avoid aggressive pre-dilation with high-pressure balloons, which can cause PR and distal embolization. Instead, low-pressure balloon pre-dilation or rotational atherectomy is preferred to achieve gentle lesion preparation while preserving plaque integrity. For lipid-rich plaques identified by OCT or NIRS, direct stenting or cautious low-pressure balloon dilation is recommended to reduce the release of plaque debris. Additionally, intravascular imaging helps to assess the true vessel diameter and lesion length, ensuring accurate pre-stent preparation that avoids under- or over-expansion—both of which can contribute to NF by damaging the microcirculation or triggering endothelial injury. For chronic total occlusion (CTO) lesions with high NF risk (e.g., long occlusion length, poor collateral circulation detected by IVUS), pre-stent administration of intracoronary thrombolytics (e.g., reteplase) may be considered to reduce the thrombus burden before stenting [96,97].

The selection of stents and balloons is also guided by intravascular imaging to reduce NF/SF risk. For patients with high NF risk (e.g., because of STEMI, large thrombus burden, vulnerable plaques), drug-eluting stents (DES) with biocompatible coatings (e.g., zotarolimus-eluting stents) are preferred over bare-metal stents (BMS). DES reduce in-stent restenosis and chronic inflammation, both of which are major factors contributing to long-term microvascular dysfunction. In cases of imaging-detected small-vessel disease or microvascular fragility, smaller-caliber stents with thinner struts are selected to minimize vessel wall trauma and preserve microcirculatory flow. For lesions with severe calcification or uneven plaque distribution, non-compliant (NC) balloons are used for pre-dilation to ensure uniform lesion expansion without excessive vessel injury. Moreover, drug-coated balloons (DCBs) may be considered for patients at high risk of NF to reduce post-procedural inflammation and endothelial dysfunction. Recent advances in intravascular imaging, such as IVUS-OCT integrated multimodal imaging, further enable the precise matching of stent/balloon size to lesion characteristics, thereby optimizing device selection and reducing NF incidence [98].

## 5. Current Limitations

Despite notable progress in intravascular imaging for predicting the coronary NF/SF phenomenon, there is still substantial room for improvement in clinical practice. This is mainly reflected in three aspects. First, most existing studies focus on the application of a single imaging modality, and there is a lack of multicenter, large-sample head-to-head comparisons of predictive efficacy between different techniques. This hinders the establishment of a unified standard for the selection of the optimal modality in clinical practice, leading to inconsistent clinical decision-making across institutions. Second, each imaging technique has inherent limitations. IVUS cannot effectively identify microthrombi or detailed intimal features, potentially missing subtle pathological changes linked to microcirculatory obstruction. OCT, despite its ultra-high resolution, has a limited penetration depth (only 1–2 mm), preventing visualization of deep vascular structures (e.g., EEM, adventitia) that are critical for assessing overall vascular remodeling. NIRS, while effective for quantifying lipid components, cannot simultaneously evaluate vascular structure or hemodynamic status, thus failing to capture the complex interaction between anatomical and functional factors underlying the NF/SF phenomenon. Therefore, reliance on a single technique cannot fully cover the multifactorial pathological mechanisms of these phenomena. Third, current predictive models are mostly based on correlation analyses between imaging features and clinical events, with insufficient exploration of the causal relationships among imaging features, pathological mechanisms, and clinical outcomes. This lack of mechanistic insight limits the generalizability and stability of the models, as they may not account for patient-specific variations in pathophysiological responses (e.g., individual differences in inflammatory reactivity or coagulation status) that affect the development of NF/SF phenomenon.

Despite the well-documented benefits of IVUS and OCT in optimizing PCI outcomes, several critical limitations hinder their universal application in routine clinical practice and ACS, particularly in STEMI settings. These limitations include their feasibility in routine and acute care settings, the cost burden, gaps in operator training, and technical challenges associated with IVUS deployment in STEMI. First, the feasibility of IVUS and OCT in routine clinical practice is constrained by significant geographic variability, workflow barriers, and inconsistent adoption, even in clinical settings where current guidelines recommend their utilization. The 2024 European Society of Cardiology (ESC) guidelines upgraded the recommendation for IVUS/OCT to Class IA for complex PCI. However, real-world clinical data indicates persistent underutilization of these imaging modalities. A 2025 debate published in *REC*:* Interv Cardiol* reported that intravascular imaging is employed in only 15% of PCI patients in Spain, despite guideline endorsement. This was primarily attributable to workflow disruptions and resource constraints [99]. In the context of ACS, although IVUS/OCT provide valuable insights into plaque morphology (e.g., erosion, rupture) and thrombus burden, their routine feasibility is limited by the urgency of reperfusion therapy, with many clinical centers prioritizing rapid revascularization over imaging-guided optimization.

Second, the cost burden associated with IVUS and OCT remains a major barrier to their widespread implementation, despite evidence supporting their long-term cost-effectiveness. Upfront procedural costs are significantly elevated due to the use of disposable catheters and extended procedural duration (average +15–20 minutes per case). A 2025 Irish micro-costing study published in *Open Heart* found that IVUS increases the baseline PCI cost (€3082) by €752, while OCT adds €884, with disposable catheters accounting for 72% of the incremental cost [100]. Global variability in cost further exacerbates this limitation. For instance, a 2024 analysis of the National Inpatient Sample (NIS) database in the United States revealed that IVUS-guided PCI is nearly twice as expensive as angiography-guided PCI ( $ 141,920 vs. $ 71,568) [101]. Although long-term cost-effectiveness is supported by reductions in repeat revascularization and stent thrombosis, the upfront financial barrier remains insurmountable in resource-constrained settings.

Third, operator training and proficiency in IVUS/OCT interpretation and deployment are uneven globally, limiting the practical utility of these imaging modalities. A 2025 survey of 1200 interventional cardiologists across 32 countries, published in *JACC Cardiovasc Interv*, found that only 41% achieved independent competence in both IVUS and OCT interpretation, with 68% reporting inadequate training during their fellowship [102]. The survey also noted that proficiency varies with clinical experience—younger cardiologists (<45 years) were more likely to utilize IVUS/OCT regularly (79%) compared to senior physicians (<30%), resulting in a generational gap in expertise.

Fourth, the deployment of an IVUS catheter during STEMI is technically challenging, particularly in cases where guidewire crossing is already difficult, further limiting its feasibility in acute clinical settings. STEMI lesions are frequently characterized by severe stenosis, thrombus burden, tortuosity, or calcification, all of which impede the advancement of IVUS catheters. Time pressure for rapid reperfusion further complicates IVUS utilization, as imaging extends the procedural duration by approximately 14 minutes, with some centers prioritizing door-to-balloon time over imaging-guided optimization [103]. Furthermore, while OCT offers higher resolution, it requires blood clearance with contrast media. This increases the contrast volume (median 320.0 mL for OCT vs. 270.0 mL for IVUS) and may elevate the risk of contrast-induced nephropathy in STEMI patients with pre-existing renal dysfunction [104].

These inherent technical drawbacks, combined with the aforementioned challenges in feasibility, cost, training, and STEMI deployment, demonstrate that IVUS and OCT are not universally easy to deploy, or even feasible in unselected routine clinical practice or unstable STEMI settings. Collectively, these limitations underscore the need for improved accessibility to training programs, cost reduction strategies, and further technical advances to fully realize the potential of IVUS/OCT in optimizing coronary revascularization outcomes.

## 6. Future Perspectives

Future advances in intravascular imaging for predicting the coronary NF/SF phenomenon are likely to focus on three core areas: multimodal fusion, intelligent integration, and personalized intervention. For multimodal fusion, combining structural imaging (IVUS/OCT) with compositional imaging (NIRS), and further integrating with functional indices like fractional flow reserve and instantaneous wave-free ratio, will establish a tripartite “anatomy-function-composition” predictive system. This addresses single-modality limitations by simultaneously capturing structural vulnerabilities (e.g., thin fibrous caps), compositional risks (e.g., high lipid burden), and functional impairment (e.g., reduced myocardial perfusion), thereby enhancing prediction accuracy and comprehensiveness. For intelligent integration, deep learning-based radiomics can extract latent predictive biomarkers from large imaging datasets, automatically identifying subtle patterns (e.g., microcalcification distribution) that are undetectable manually and allowing the development of automated models. These models enable rapid risk stratification of the NF/SF phenomenon, providing real-time data-driven support during PCI and optimizing procedural efficiency [105]. In the future, it may be feasible to integrate radiomic features derived from OCT/IVUS (e.g., plaque texture, calcification distribution) with patient clinical data (e.g., age, diabetes history) and genetic markers (e.g., ApoE gene polymorphism) to construct an artificial intelligence-based, personalized model for predicting the risk of NF/SF phenomenon, thus improving the accuracy of risk stratification. Moreover, automated image analysis algorithms can be developed to achieve real-time identification of high-risk imaging features (e.g., TCFA, PR), providing immediate guidance for interventional treatment decisions. For personalized intervention, future studies should clarify causal links between imaging biomarkers and NF/SF phenomenon, validate new predictive models via prospective randomized controlled trials across diverse populations (e.g., diabetic patients) and scenarios (e.g., primary PCI for STEMI vs. elective PCI for stable CAD), and explore imaging-guided personalized strategies (e.g., rotational atherectomy for heavy calcification, tailored antithrombotic regimens for high-lipid lesions) to advance from risk prediction to precision prevention and management [106].

## 7. Conclusion

With the relentless progression of technological innovation and the expansion of in-depth clinical research initiatives, intravascular imaging will undoubtedly become an increasingly core tool in optimizing the clinical care of patients with the coronary NF/SF phenomenon. Ultimately, this will establish a more solid foundation for improving the prognostic outcomes of individuals suffering from CAD.
